# Acute Transverse Myelitis in a Patient With Type 2 Loeys-Dietz Syndrome: A Report of a Rare Case From India

**DOI:** 10.7759/cureus.65524

**Published:** 2024-07-27

**Authors:** Swati Parida, Abhishek Pathak, Vijaya Nath Mishra

**Affiliations:** 1 Department of Neurology, Kalinga Institute of Medical Sciences, Bhubaneswar, IND; 2 Department of Neurology, Institute of Medical Sciences, Banaras Hindu University, Varanasi, IND

**Keywords:** bifid uvula, skeletal deformities, transforming growth factor-β, transverse myelitis, loeys–dietz syndrome

## Abstract

Loeys-Dietz syndrome (LDS) is a very rare connective tissue disorder with autosomal dominant inheritance, characterized by the involvement of the cardiovascular, musculoskeletal, and cutaneous systems, along with dysmorphic facial features. Currently, there are limited data regarding this disease. This case presents a clinical observation of a 17-year-old boy with acute onset of sensorimotor paraparesis and genetically confirmed LDS. The predominant symptoms of LDS include arterial aneurysms, arterial tortuosity, hypertelorism, and bifid uvula. However, this constellation of symptoms is not found in all patients with the disease. Genetic confirmation is essential for an accurate diagnosis. The prognosis for LDS differs from its mimics, such as Marfan syndrome, Beals syndrome, Ehlers-Danlos syndrome, and Shprintzen-Goldberg syndrome. Management of the disease warrants a multidisciplinary approach to address its various manifestations. Such an approach can help increase the life expectancy and improve the quality of life for these patients.

## Introduction

Loeys-Dietz syndrome (LDS) is a connective tissue disorder with autosomal dominant inheritance [1]. Mutations in genes encoding transforming growth factor beta-1/2 (*TGFBR1* and *TGFBR2*), as well as decapentaplegic homolog-3 (*SMAD3* and *TGFB2*), cause LDS [1,2]. LDS is characterized by vascular abnormalities (cerebral, thoracic, and abdominal aorta aneurysms/dissections), skeletal deformities (pectus excavatum/carinatum, scoliosis, joint laxity, arachnodactyly, talipes equinovarus, cervical spine malformation), dysmorphic craniofacial features (hypertelorism, strabismus, bifid uvula/cleft palate, and craniosynostosis), and dermatological manifestations (velvety and translucent skin, easy bruising, and dystrophic scars). LDS is diagnosed by characteristic clinical features in the proband and family members and/or by the presence of a heterozygous pathogenic variant in *SMAD2*, *SMAD3*, *TGFB2*, *TGFB3*, *TGFBR1*, or *TGFBR2 *[3].

Type 1 LDS is due to mutations in genes encoding *TGFBR1* (9q22.33), and type 2 LDS is due to mutations in genes encoding *TGFBR2 *(3q24.1). Type 3 LDS, type 4 LDS, type 5 LDS, and type 6 LDS are due to mutations in genes encoding *SMAD3* (15q22.33), *TGFB2 *(1q41), *TGFB3 *(14q24.3), and *SMAD2*, respectively [3]. We hereby report an interesting case of acute transverse myelitis with genetically proven LDS.

## Case presentation

A 17-year-old Indian boy presented to the Neurology clinic with acute onset sensorimotor paraparesis, along with bowel and bladder involvement, for one day. The weakness of both lower limbs rapidly progressed over 24 hours. On the following day, he was unable to move his lower limbs and experienced urinary retention. There was no history of recent vaccination, insect or animal bite, fever, multiple joint pain, photosensitivity, cough, oral or genital ulcers, rashes, diarrhea, excessive daytime sleepiness, headache, seizures, nausea, or vomiting. He did not have any weakness of the neck or upper limbs, respiratory difficulty, craniobulbar deficits, visual problems, or altered sensorium.

**Figure 1:**
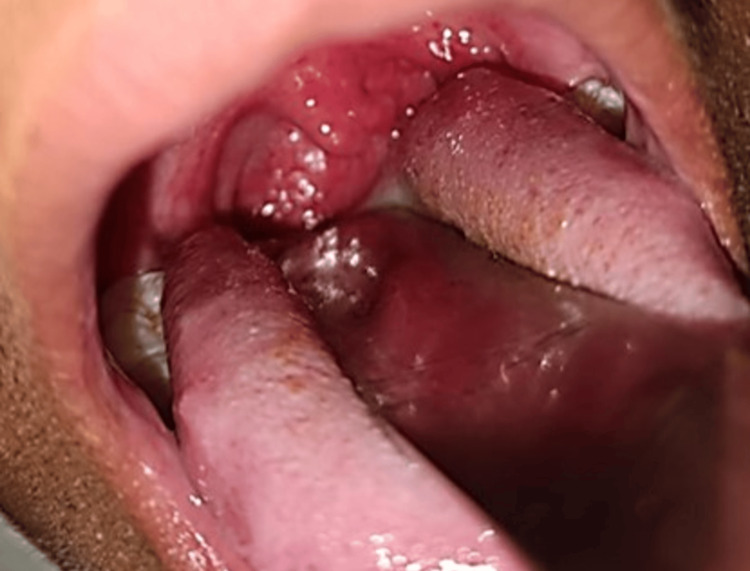
The patient has a bifid and broad uvula.

**Figure 2:**
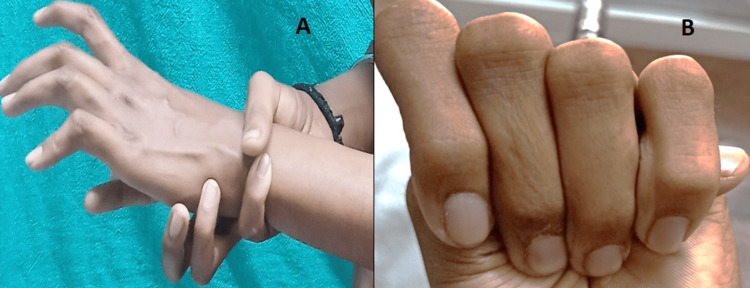
(A) Positive wrist sign. (B) Positive thumb sign.

**Figure 3:**
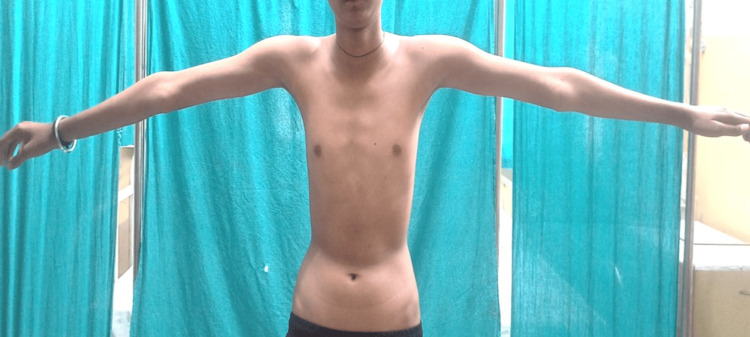
The arm span of the patient is more than his height; marfanoid habitus is present.

**Figure 4:**
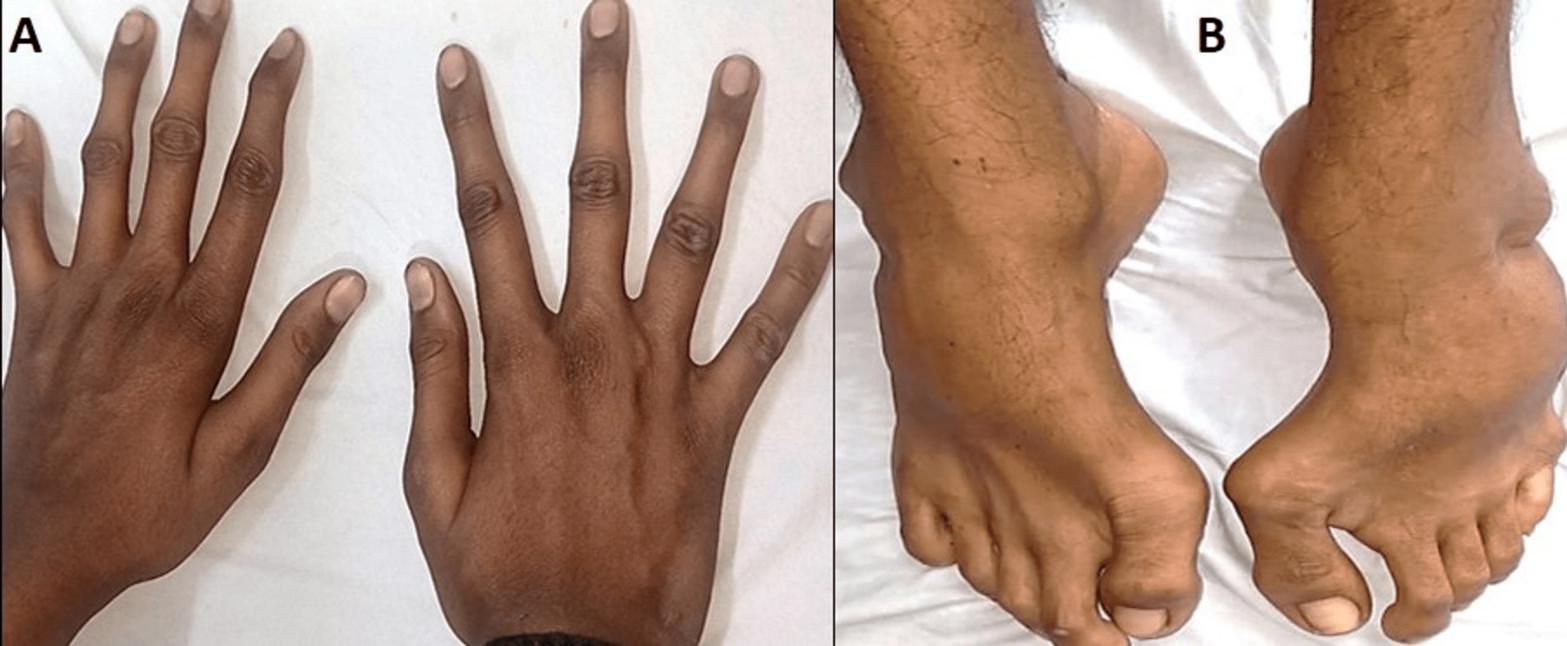
(A) Arachnodactyly of fingers. (B) Camptodactyly of toes with contracture.

On clinical examination, he had hypertelorism, divergent squint, dolichocephaly, a bifid uvula (Figure 1), retrognathia, joint laxity (positive wrist and thumb sign) (Figure 2A, 2B), marfanoid habitus (Figure 3), arachnodactyly and camptodactyly of toes (Figure 4A, 4B), and fixed contractures of the ankle joint. His vitals were stable. On examination of the central nervous system (CNS), the power in both lower limbs was Medical Research Council (MRC) grade 0/5, while the power in the upper limbs was 5/5. Sensory examination revealed a 60% loss of pain and temperature sensations below the T10 level. Vibration sensation was impaired below the T10 dermatome. The abdominal reflex was absent. Deep tendon reflexes were absent in both lower limbs and 2+ in the upper limbs. Bilateral plantar reflexes were absent. The rest of the CNS examination was normal. Respiratory, abdominal, and cardiovascular system examinations were normal.

**Figure 5:**
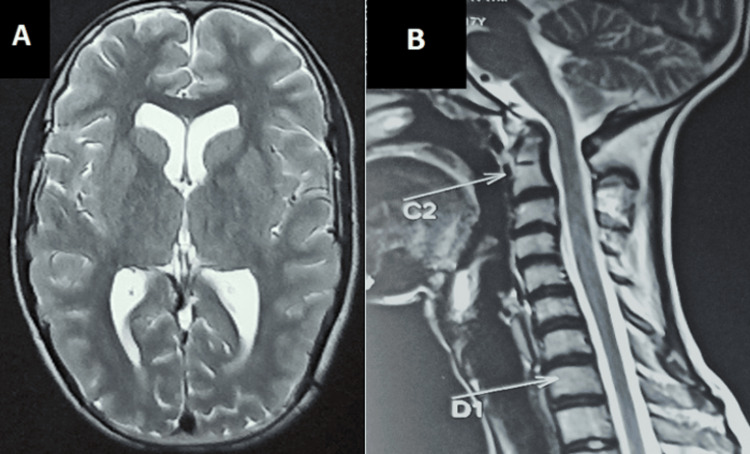
(A) MRI of the brain revealed a dolichocephalic skull with a decreased cephalic index. (B) Screening MRI of the whole spine revealed atlanto-axial subluxation with mild retroflexion of the dens, indenting the anterior subarachnoid space at the craniovertebral junction.

**Figure 6:**
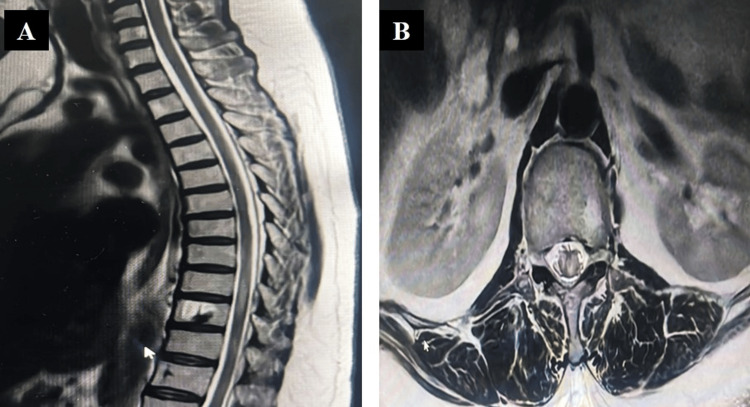
(A) MRI of the dorsolumbar spine revealed myelitis at D9-10 levels in the sagittal section. (B) Myelitis evident in the axial section.

Baseline investigations, which included a complete hemogram, renal function test, liver function test, lipid profile, thyroid profile, and serum electrolytes, were normal. Viral markers for HIV, hepatitis B and C, and VDRL were negative. Ultrasound of the abdomen and pelvis, electrocardiogram, echocardiography, and fundoscopy were normal. Magnetic resonance imaging (MRI) of the brain and dorsolumbar spine, along with screening of the whole spine with contrast, revealed a dolichocephalic skull with a decreased cephalic index and atlanto-axial subluxation with mild retroflexion of the dens indenting the anterior subarachnoid space at the craniovertebral junction (Figure 5A, B) and myelitis at the D9-10 level (Figure 6A, B).

Serology for antibodies to aquaporin-4, myelin-oligodendrocyte glycoprotein, nuclear, neutrophil cytoplasmic, and anti-phospholipid antigens was negative.

Cerebrospinal fluid (CSF) examination was normal (WBC count: 4 cells/μL, sugar: 50 mg/dL, corresponding blood sugar: 93 mg/dL, protein levels: 40.5 mg/dL). He was diagnosed with postinfectious acute transverse myelitis at the T10 level. We report this case as, to our surprise, he had other features of a connective tissue disorder (Figures 1-4). Computed tomography angiography (CTA) of the aorta and its branches did not show any aneurysms. Diagnostic spinal angiography was performed to rule out any spinal arterial aneurysm, dural ectasia, or dissection that could lead to transverse myelitis, but it was normal. He was treated with intravenous pulse methylprednisolone (1000 mg for 5 days), which was tapered over the next three months using oral prednisolone (reducing tail was used). After four months, the weakness of both legs improved to MRC grade 4. He was able to walk without assistance. Whole exome analysis revealed a heterozygous missense variant in the *TGFBR2* gene (Chrm.3:30732948:t:CNM-003242.5, exon 7, variant- c.1561T>Cp.Trp521Arg) suggestive of LDS 2.

## Discussion

Herein, we report the case of a 17-year-old boy who presented with acute transverse myelitis and features of LDS. LDS is characterized by an unfavorable prognosis. The average life expectancy of LDS patients varies from 26 to 37 years, according to various reports [1,4]. Fatal outcomes are discernible in LDS cases with dissection/rupture of the aneurysms of the aorta or large-caliber arteries and intracranial hemorrhages [4]. Aortic root aneurysms are present in 98% of LDS patients, but aneurysms can also occur in other arteries such as the coronary artery, pulmonary artery, ductus, and subclavian arteries [5]. TGF-beta signaling plays a critical role in vital cellular processes such as embryogenesis, tissue homeostasis, cell differentiation, inflammation, and vascular remodeling. Genetic mutations result in the activation of the TGF-beta pathways, leading to the degradation of the extracellular matrix and increased susceptibility to aortic dilatation, dissection, and other clinical features of LDS [6]. Aortic root aneurysms are present in two-thirds of LDS patients, and around one-fifth of LDS patients with an established diagnosis have an aortic dissection [7]. LDS-1 and LDS-2 patients with severe cranial and facial features are particularly vulnerable to ruptures at early ages and at smaller dimensions compared to those with other aneurysm syndromes like Marfan and Ehlers-Danlos syndrome [1,2]. In our patient, aneurysms of the aorta or its branches were absent. Although our patient did not have any aortic root dilatation or dissection, regular echocardiography is needed to monitor its presence, as it is important to treat it at the earliest.

Craniofacial anomalies are more common in Type 1 LDS, whereas these are largely absent in Type 2 LDS. Type 2 LDS is characterized by predominant skin involvement, causing increased susceptibility to bruising, abnormal scarring, and transparent skin [8]. Our patient was a genetically confirmed case of LDS type 2 and had craniofacial anomalies.

Various neurological manifestations of LDS include learning disabilities (seen more commonly in LDS-1 and LDS-2, and if present, usually related to craniosynostosis or hydrocephalus), intellectual disabilities in LDS-4, Chiari malformation, hydrocephalus, headache, and dural ectasia [9-11]. Differential diagnoses of LDS include Marfan syndrome, Beals syndrome, Ehlers-Danlos syndrome, and Shprintzen-Goldberg syndrome [12]. The cause of the dorsal myelitis was not known in our patient, and whether it was due to the disease per se or other causes could not be confirmed.

## Conclusions

Our case presents a common disease presentation, i.e., transverse myelitis, associated with an uncommon and rare disease, namely LDS. The data regarding LDS are limited, so we should report the various findings and manifestations of this rare disease to enhance our understanding and improve patient care. We need to report and follow up on all such cases.
